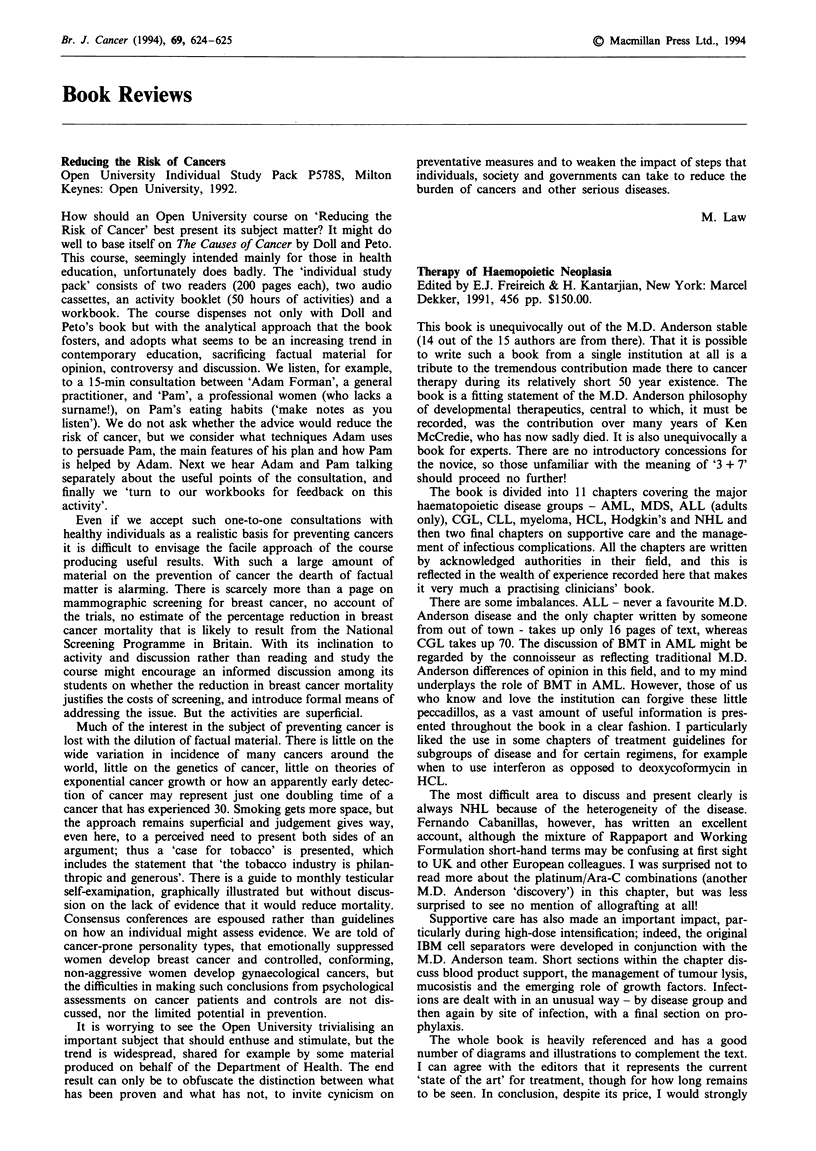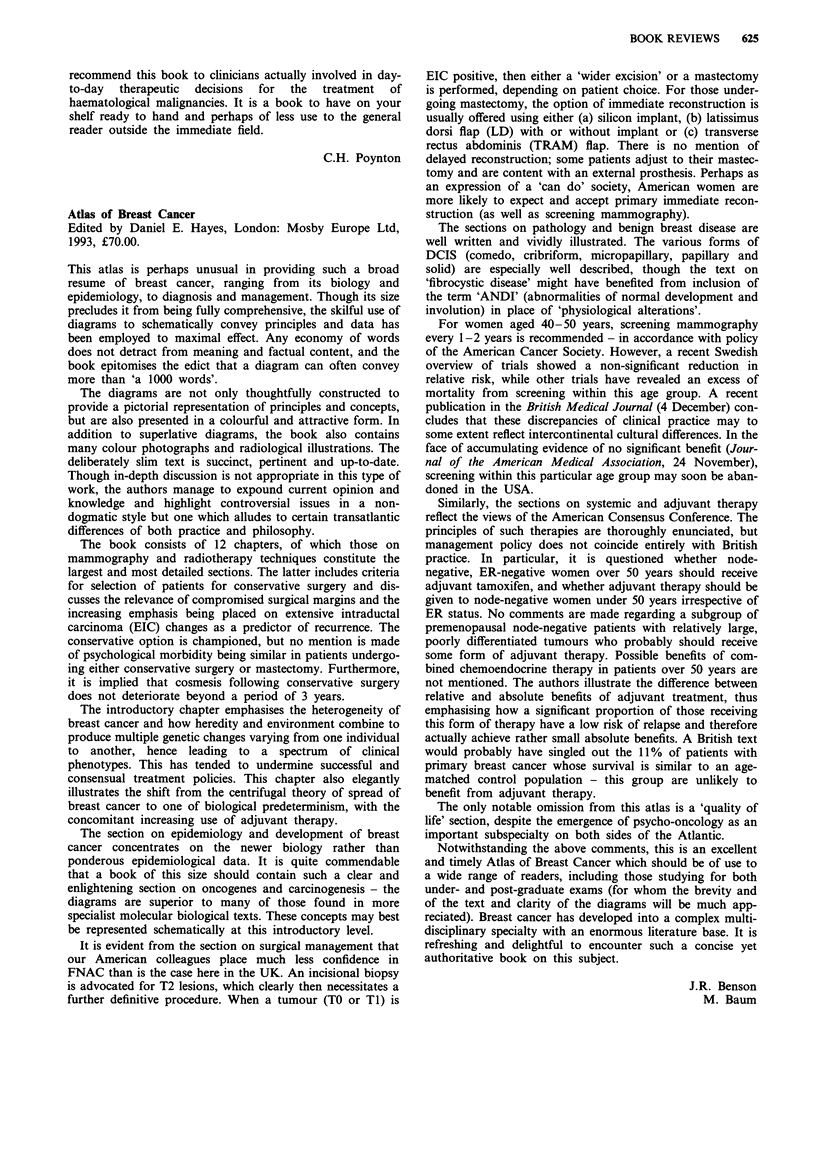# Therapy of Haemopoietic Neoplasia

**Published:** 1994-03

**Authors:** C.H. Poynton


**Therapy of Haemopoietic Neoplasia**
Edited by E.J. Freireich & H. Kantarjian, New York: Marcel Dekker, 1991, 456 pp. $150.00.

This book is unequivocally out of the M.D. Anderson stable (14 out of the 15 authors are from there). That it is possible to write such a book from a single institution at all is a tribute to the tremendous contribution made there to cancer therapy during its relatively short 50 year existence. The book is a fitting statement of the M.D. Anderson philosophy of developmental therapeutics, central to which, it must be recorded, was the contribution over many years of Ken McCredie, who has now sadly died. It is also unequivocally a book for experts. There are no introductory concessions for the novice, so those unfamiliar with the meaning of '3 + 7' should proceed no further!

The book is divided into 11 chapters covering the major haematopoietic disease groups - AML, MDS, ALL (adults only), CGL, CLL, myeloma, HCL, Hodgkin's and NHL and then two final chapters on supportive care and the management of infectious complications. All the chapters are written by acknowledged authorities in their field, and this is reflected in the wealth of experience recorded here that makes it very much a practising clinicians' book.

There are some imbalances. ALL - never a favourite M.D. Anderson disease and the only chapter written by someone from out of town - takes up only 16 pages of text, whereas CGL takes up 70. The discussion of BMT in AML might be regarded by the connoisseur as reflecting traditional M.D. Anderson differences of opinion in this field, and to my mind underplays the role of BMT in AML. However, those of us who know and love the institution can forgive these little peccadillos, as a vast amount of useful information is presented throughout the book in a clear fashion. I particularly liked the use in some chapters of treatment guidelines for subgroups of disease and for certain regimens, for example when to use interferon as opposed to deoxycoformycin in HCL.

The most difficult area to discuss and present clearly is always NHL because of the heterogeneity of the disease. Fernando Cabanillas, however, has written an excellent account, although the mixture of Rappaport and Working Formulation short-hand terms may be confusing at first sight to UK and other European colleagues. I was surprised not to read more about the platinum/Ara-C combinations (another M.D. Anderson 'discovery') in this chapter, but was less surprised to see no mention of allografting at all!

Supportive care has also made an important impact, particularly during high-dose intensification; indeed, the original IBM cell separators were developed in conjunction with the M.D. Anderson team. Short sections within the chapter discuss blood product support, the management of tumour lysis, mucosistis and the emerging role of growth factors. Infections are dealt with in an unusual way - by disease group and then again by site of infection, with a final section on prophylaxis.

The whole book is heavily referenced and has a good number of diagrams and illustrations to complement the text. I can agree with the editors that it represents the current 'state of the art' for treatment, though for how long remains to be seen. In conclusion, despite its price, I would strongly recommend this book to clinicians actually involved in day-to-day therapeutic decisions for the treatment of haematological malignancies. It is a book to have on your shelf ready to hand and perhaps of less use to the general reader outside the immediate field.

C.H. Poynton